# A novel prognostic scoring system HATS for acute myeloid leukemia patients undergoing allogeneic hematopoietic stem cell transplantation

**DOI:** 10.1007/s00277-026-06989-z

**Published:** 2026-04-09

**Authors:** Garret M K Leung, Yishan Ye, Yi Luo, Jimin Shi, Yanmin Zhao, Joycelyn P.Y. Sim, Yok-Lam Kwong, Huang He, Harinder Gill

**Affiliations:** 1 Department of Medicine, School of Clinical Medicine, LKS Faculty of Medicine, University of Hong Kong, Hong Kong, China; 2 Bone Marrow Transplantation Center, The First Affiliated Hospital, Zhejiang University School of Medicine, Hangzhou, China

**Keywords:** acute myeloid leukemia, allogeneic hematopoietic stem cell transplantation, prognostic model

## Abstract

The optimal prognostication model for outcome following allogeneic hematopoietic stem cell transplantation (allo-HSCT) for acute myeloid leukemia (AML) remains undefined. To establish a prognostic scoring system, 466 consecutive AML patients undergoing allo-HSCT from 2014 to 2023 were analyzed as a training cohort. Six donor, leukemia and recipient related factors were identified as prognostically significant and assigned weighted scores, respectively 1 point each for donor cytomegalovirus seropositivity, Hemopoietic Cell Transplantation-specific Comorbidity Index ≥ 2, secondary AML, not in first complete remission, and low-intensity induction therapy; 3 points for inv(3)/t(3;3)(q21;q26) or *KMT2A* rearrangement; 4 points for − 5/del(5q) or *TP53* mutation; 0 point for *CEBPA* bZIP in-frame mutation or core-binding factor AML; and 1 point for all other genetic risk groups. Scores were grouped into four risk categories: favorable (0–1), intermediate (2–3), poor (4–5), and very poor (≥ 6); constituting the HATS prognostication model. The corresponding 2-year overall survivals of favorable, intermediate, poor and very poor risk groups were 92%, 78%, 50%, and 7.4% (log-rank *P* < 0.001). An external validation cohort comprising 395 patients was similarly analyzed. With concordance statistics, HATS outperformed all six existing prognostication models. In conclusion, HATS represents a novel prognostication model devised for AML patients undergoing allo-HSCT.

Clinicaltrials.gov identifier: NCT06702111.

**Supplementary Information:**

The online version contains supplementary material available at 10.1007/s00277-026-06989-z.

## Introduction

Acute myeloid leukemia (AML) is a heterogenous disease with diverse outcomes [1, 2]. Although allogeneic hematopoietic stem cell transplantation (allo-HSCT) is a potentially curative therapy, it is associated with significant morbidities and mortality [3, 4]. Accurate prognostication is therefore critical for guiding treatment decisions, patient counselling, resource allocation, improving transplantation outcomes, and identifying clinical needs for future research.

Numerous prognostication models based on a wide array of clinical variables have been developed in the past two decades to predict outcome following allo-HSCT [5–11]. However, the optimal prognostic scoring system remains undefined, due to the complicated interplay between patient, leukemia, and transplantation factors [12–14]. The European LeukemiaNet (ELN) 2022 classification is limited in its ability to predict outcome after allo-HSCT, as it relies solely on genomic risks and is primarily geared towards adult patients younger than 60 years treated with intensive induction therapy [15]. Efforts to improve ELN 2022 include re-classifying its subgroups and incorporating measurable residual disease (MRD) into the risk assessment [16–18]. The hematopoietic cell transplantation comorbidity index (HCT-CI) was developed nearly two decades ago based on the Charlson Comorbidity Index, which was used to predict one-year mortality in patients admitted to general medical centers [5]. Although the ability of HCT-CI to predict post allo-HSCT survival was validated subsequently, these studies all grouped scores of 1 and 2 together, limiting its ability to further subclassified patients with fewer comorbidities [19–21, 5, 22]. Similarly, an HCT-CI cut-off of ≥ 3 is also used in existing prognostication models [10, 11]. While HCT-CI only relies on comorbidities, the disease-risk index (DRI) solely considers the cytogenetic risk and remission status at transplantation, and is intended for studying outcomes across broad disease categories [7]. Notably, the DRI cytogenetic risk is mainly based on the CIBMTR grouping published more than a decade ago in 2012. This grouping defines favorable-risk as core-binding factor (CBF)-AML only, adverse-risk as complex cytogenetics with ≥ 4 abnormalities, and intermediate-risk as others; [23, 7] which obviously is very different from current cytogenetic risk categorization [15]. The disease risk comorbidity index (DRCI) divides patients into two subgroups based on HCT-CI (< 3 *versus* ≥ 3), resulting in a total of six risk categories [10]. The AML-specific disease risk group (AML-DRG) and AML hematopoietic cell transplant-composite risk (AML-HCT-CR) integrate MRD (flow cytometric or molecular) in the risk stratification, with ELN 2017 used for genetic risk assessment, and HCT ≥ 3 and age ≥ 60 years as additional risk factors [11]. Importantly, unknown MRD is treated as positive in these two models, with over 40% of patients in the original study having unknown MRD [11]. Such limitations affect the inclusion of MRD status in prognostication, especially in cohorts with incomplete MRD information.

To address the lack of a model dedicated to predicting outcome of AML patients undergoing allo-HSCT, we studied a retrospective series of patients with currently available models to ascertain their prognostic powers, and then with a panel of donor, recipient and leukemia factors to determine relevant prognostic markers. The objective was to use this patient series as a training cohort to establish a novel prognostication model, which would then be validated with an external cohort.

## Materials and methods

### Training cohort 

Consecutive adult patients with AML undergoing allo-HSCT at Queen Mary Hospital (QMH), Hong Kong, China, between January 1, 2014 and December 31, 2023 were retrospectively analyzed and constituted the training cohort. Decisions on allo-HSCT were based on leukemia factors (risk stratification, remission status, MRD) and patient factors (performance score, HCT-CI, and donor availability). Details of indications for allo-HSCT and patient selection were given in Supplementary Table 1. This study was approved by the Institutional Review Board of the University of Hong Kong (UW 24–621) and registered at clinicaltrials.gov (identifier: NCT06702111) and conducted in accordance to the Declaration of Helsinki.

### Allo-HSCT

 Conditioning regimens were categorized as myeloablative or reduced intensity according to American Society of Transplantation and Cellular Therapy (ASTCT) criteria [24]. Graft versus host disease (GVHD) prophylaxes were post-transplantation cyclophosphamide (PTCy) in haploidentical and selected mismatched unrelated (MMUD) transplantations, and conventional calcineurin inhibitor-based in matched sibling, matched unrelated, and other MMUD transplantations. All patients received standard antimicrobial prophylaxis and supportive care according to institutional protocols.

### MRD testing

MRD assessment was performed in specific subsets of patients. *NPM1* and *CBF::MYH11* MRD was detected by in-house droplet digital polymerase chain reaction (ddPCR) with a sensitivity of 10^− 5^ to 10^− 6^. *RUNX1::RUNX1T1* MRD was detected with an in-house reverse transcription PCR, with a sensitivity of 10^− 5^ to 10^− 6^. A minority of patient had case-specific MRD assessment by ddPCR based on other gene mutations or fusions with a sensitivity of 10^− 4.5^ to 10^− 5^ [25]. 

### Validation cohort

Consecutive adult patients with AML undergoing allo-HSCT at the First Affiliated Hospital, Zhejiang University School of Medicine, Hangzhou, China, between January 1, 2018 and December 31, 2021 were retrospectively analyzed and constituted the validation cohort. The indications for allo-HSCT aligned with those of QMH. This study was approved by the Ethics Committee of Clinical Trial of the First Affiliated Hospital of Zhejiang University (IIT20200086C-R1) and conducted in accordance to the Declaration of Helsinki.

### Statistical methods

The prognostic power of six existing prognostication models, including ELN 2022 [15], HCT-CI [5], DRI [7], DRCI [10], AML-DRG and AML-HCT-CR [11], was evaluated with concordance (C) statistics. A C-statistic of 1.0 indicated perfect predictive accuracy, while a value of 0.5 indicated random chance. The bootstrap method employing 1000 re-samples was used to obtain the optimism-adjusted C-statistic and 95% confidence interval (CI). The C-statistics of different models were compared with a non-parametric approach [26]. Area under time-dependent receiver operating characteristic curve (AUC) was estimated with the inverse probability of censored weighting approach [27]. Time-dependent prediction error was evaluated using integrated Brier scores (IBS), applying inverse probability of censoring weighting. All analyses were performed with the complete-case method without data imputation. Overall survival (OS), defined as the time from allo-HSCT to death (event) or last follow-up (censor), was estimated with the Kaplan-Meier method, and potential impacting factors were compared with the log-rank test. Hazards ratios (HRs) were calculated with the Cox proportional hazards regression model. Two-tailed P-values of < 0.05 were considered statistically significant. Statistical analyses were conducted with the rms, compareC, timeROC, and pec packages from the R-4.4.1 statistical software (http://cran.r-project.org/).

### Prognostic model development

In the development of a novel prognostic model, cox proportional hazards regression model was used to identify clinicopathologic, cytogenetic and molecular features predictive of survivals after allo-HSCT in the training cohort. Significant variables on univariate and multivariate analyses were retained to obtain the β coefficients. Disease risk scores were calculated from weighted points according to the β coefficients (1 point for β at ≤ 1.0; 2 points for β at 1.0–1.5; 3 points for β at 1.6–2.0; and 4 points for β at > 2). The total score was the sum of points from individual risk factors. The overall scores were grouped into 4 risk categories (favorable, intermediate, poor, very poor) based on their association with OS. In turn, the prognostic significance of each significant variable and risk categories of the novel prognostic model was tested on the validation cohort with the cox proportional hazard model and C statistic.

## Results

### Training cohort

 Four hundred and sixty-six consecutive patients (men, *N* = 211; women, *N* = 255) with a median age of 51 (18–68) years were analyzed (Table 1). The majority (87%) of patients received intensive chemotherapy for remission induction. Patients who received low-intensity induction, based on risk/benefit considerations, were significantly older, and had poorer (intermediate/adverse) ELN 2022 risk diseases that were relapsed or refractory (Supplementary Table 2). Most patients (*N* = 368, 79%) had HCT-CI = 0 and hence few pre-transplantation comorbidities. Pre-HSCT, most patients were in complete remission (CR) (CR1, *N* = 307, 66%; CR2, *N* = 140, 30%; ≥CR3, *N* = 13, 3%), with only six patients (1%) in non-remission (NR).

**Table 1:** Clinicopathologic features of the training cohort of 466 patients with acute myeloid leukemia (AML) undergoing allogeneic hematopoietic stem cell transplantation (HSCT)

Clinicopathologic parameters	Numbers
Age	
Recipient	
Median – years (range)	51 (18-68)
≥60 years – number (%)	68 (15%)
Donor	
Median – years (range)	38 (15-67)
≥35 years – number (%)	271 (58%)
Male sex – number (%)	
Recipient	211 (45%)
Donor	265 (57%)
CMV seropositivity – number (%)	
Recipient	418 (90%)
Donor	328 (70%)
HCT-CI – number (%)	
0	368 (79%)
1	64 (14%)
≥2	34 (7%)
White cell count at diagnosis	
Median – per microliter (range)	12,000 (100-450,300)
≥20,000 per microliter – number (%)	194 (42%)
Secondary AML – number (%)	43 (9%)
Disease status at transplantation – number (%)	
CR/CRi	460 (99%)
CR1	307 (66%)
CR2	140 (30%)
CR3+	13 (3%)
NR	6 (1%)
ELN 2022 risk classification – number (%)	
Favorable	97 (21%)
Intermediate	218 (47%)
Adverse	151 (32%)
Remission induction intensity – number (%)	
Intensive	405 (87%)
Low-intensity	61 (13%)
MRD status before transplantation – number (%)	
Not available	382 (82%)
Available	84 (18%)
Positive	65 (77%)
Negative	19 (23%)
Prior allogeneic HSCT – number (%)	22 (5%)
Donor type – number (%)	
Matched sibling	177 (38%)
Matched unrelated	108 (23%)
Mismatched unrelated	67 (14%)
Haploidentical	114 (24%)
HLA matching – number (%)	
Matched	285 (61%)
≥1 HLA mismatched	181 (39%)
Hematopoietic stem cell source – number (%)	
Peripheral blood	349 (75%)
Bone marrow	117 (25%)
Conditioning intensity – number (%)	
Myeloablative	273 (59%)
Reduced intensity	193 (41%)
GVHD prophylaxis – number (%)	
Conventional	327 (70%)
PTCy-based	139 (30%)

*CMV* cytomegalovirus, *HCT-CI* hematopoietic cell transplantation comorbidity index, *CR* complete remission, *CRi* complete remission with incomplete count recovery, *NR* non-remission, *HLA* human leukocyte antigen, *MRD* measurable residual disease, *GVHD* graft-versus-host disease, *PTCy* posttransplantation cyclophosphamide

### Results of existing prognostication models in the training cohort

**Fig. 1:**
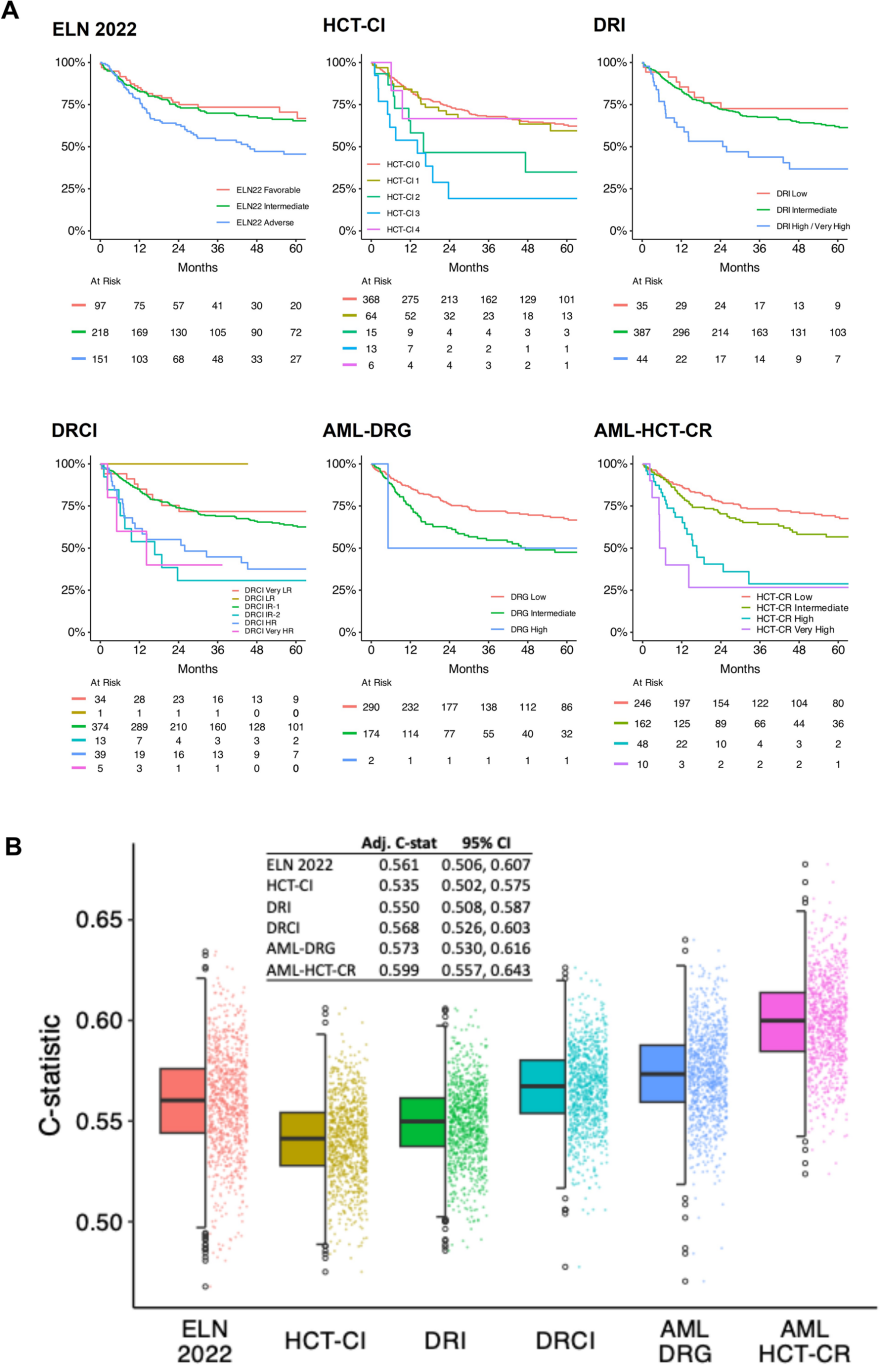
Performance of six current prognostication models in predicting post-allogeneic hematopoietic stem cell transplantation overall survival in 466 patients with acute myeloid leukemia (AML). **A.** Overall survival analysed by Kaplan-Meier method in different prognostication models. All six models showed overlapping survival curves particularly in the low and low/intermediate risk groups. **B**. Comparison of C-statistics of different prognostic models, with AML HCT-CR achieving the highest value. ELN: European LeukemiaNet; HCT-CI: Hematopoietic Cell Transplantation-Specific Comorbidity Index; DRI: Disease Risk Index; DRCI: Disease Risk Comorbidity Index; AML-DRG: Acute Myeloid Leukemia-specific Disease Risk Group; AML-HCR-CR: AML Hematopoietic Cell Transplant-composite Risk; LR: low risk; IR-1: intermediate risk-1; IR-2: intermediate risk-2; HR: high risk; C: concordance; Adj. C-stat: adjusted C-statistic; CI: confidence interval

 The results of stratification according to existing prognostication models were shown in Supplementary Table 3. Only ELN 2022 had an even risk distribution (favorable: 21%; intermediate: 47%; adverse: 32%). Other models showed significant skewing with predominant clustering of cases to one/two low-risk categories (HCT-CI, 0: 79%; DRI, intermediate: 83%; DRCI, intermediate-1: 80%; AML-DRG, low/intermediate: 99%; AML-HCT-CR, low/intermediate: 88%); suggesting that prognostic discrimination was limited particularly in the low/intermediate risk groups. Kaplan-Meier analyses of OS showed similar findings, with largely overlapping survival curves and hence poor discrimination in the low/intermediate risk categories (Fig. 1A). Accordingly, the adjusted C statistics of these models were also of low values, reflecting low discriminative power (Fig. 1B).

### Genetic/cytogenetic prognostic groups in the training cohort

 Because ELN 2022 showed better distribution of prognostic groups, selected genetic/cytogenetic changes from ELN 2022 commonly found in the training cohort were used for analysis of OS post-HSCT. Four clusters were observed (Supplementary Fig. 1). Patients with CBF AML and bZIP in-frame mutations in *CEBPA* (*CEBPA* bZIP) showed the most favorable outcome, forming the first cluster. Patients with inv(3)(q21;q26)/t(3;3)(q21;q26) and *KMT2A* rearrangements had poorer outcomes, placing them in the third cluster. Patients with *TP53* mutations (*TP53*^mut^) and − 5/del(5q) had the worst survival, forming the fourth cluster. Patients with other genetic/cytogenetic aberrations showed intermediate outcomes, forming the second cluster.

### Development of a novel prognostic model 

**Fig. 2:**
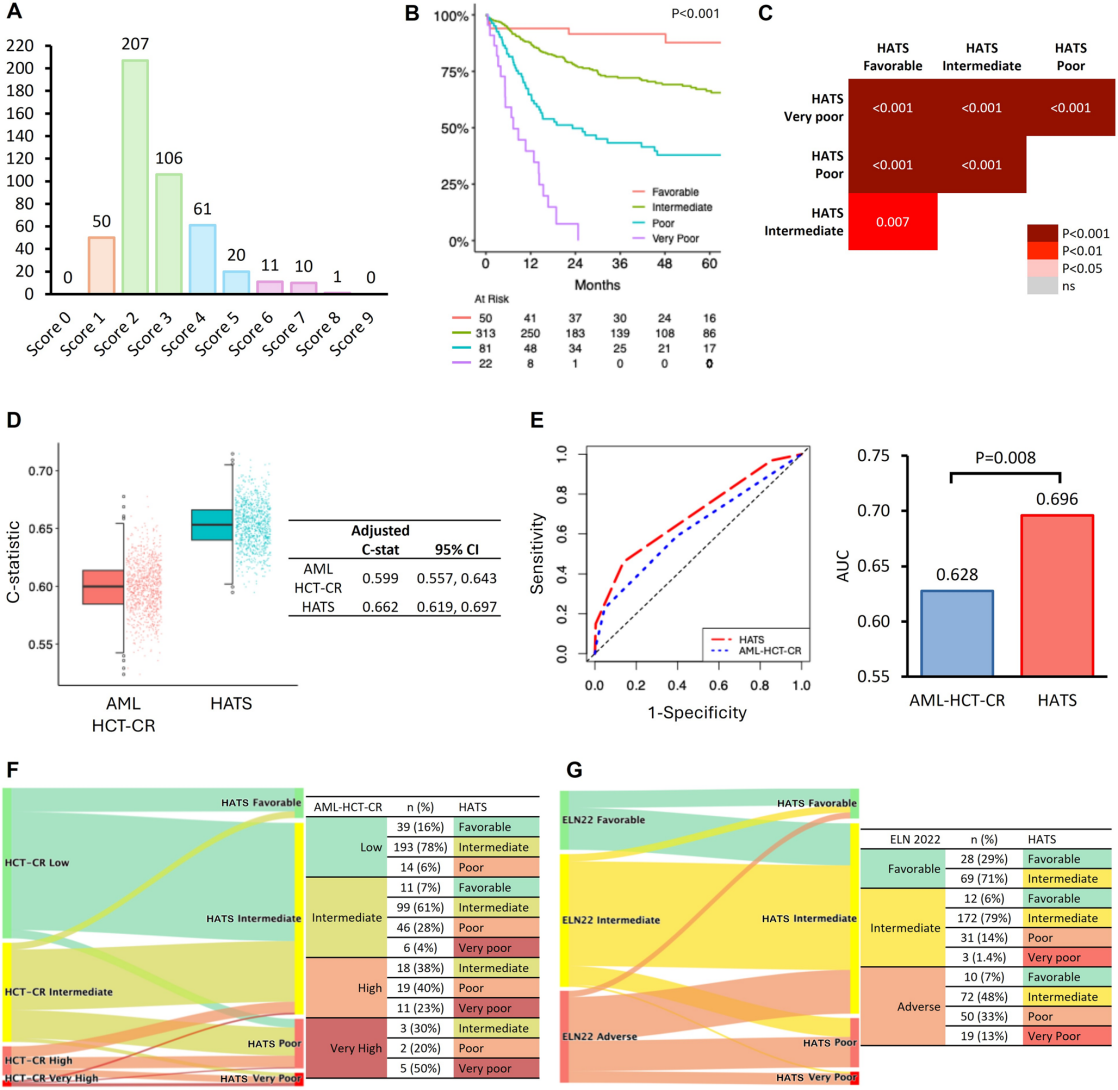
Development and performance of the novel Hong Kong Allogeneic HSCT Risk Score (HATS). **A**. Risk score distribution, with score of 2 occurring in the largest number of cases. **B**. Overall survival by HATS risk groups, showing discrete separation of survival curves between the four risk groups. **C**. Pairwise log-rank tests with adjusted p-values, showing significant differences between all four risk groups. **D**. HATS outperformed the Acute Myeloid Leukemia Hematopoietic Cell Transplant-composite Risk (AML-HCT-CR) in adjusted C-statistic. **E**. Time-dependent receiver operating characteristic curve (left) and area under time-dependent receiver operating characteristic curves (AUC) (right) of AML-HCT-CR and HATS, showing a significant difference. **F**. Re-stratification of risk groups from European LeukemiaNet (ELN) 2022 to HATS. **G**. Re-stratification of risk groups from AML-HCT-CR to HATS.

The impacts on OS of the genetic/cytogenetic grouping and eighteen patient-, disease- and treatment-related clinicopathologic parameters, selected based on their reported prognostic relevance in AML after allo-HSCT, were evaluated by Cox regression analyses (Table 2). On multivariate analysis, factors associated with inferior OS included donor cytomegalovirus (CMV) seropositivity, HCT-CI score ≥ 2, secondary AML, non-CR1 (≥ CR2 and NR) at HSCT, genetic risk groups other than *CEBPA* bZIP or CBF AML, and prior low-intensity induction therapy. Weighted points were assigned to each significant variable in proportion to the β coefficients obtained in the final multivariate model (Table 3). The aggregate score was referred to as the *H*ong Kong *a*llogeneic HSC*T* risk *s*core (HATS), with possible values of 0–9. The HATS scores of the training cohort ranged from 1 to 8, with 2 as the most common score (*N* = 207, 44%), followed by 3 (*N* = 106, 23%) and 4 (*N* = 61, 13%) (Fig. 2A). Kaplan-Meier analysis showed progressively decreasing OS with increasing HATS scores, with 1 portending the best and ≥ 6 portending the worst survivals (Supplementary Fig. 2). Scores with HRs less than 2 versus the reference group were classified as favorable, scores with HRs from 2 to less than 5 as intermediate; scores with HRs from 5 to less than 15 as poor; and scores with HRs of 15 or higher as very poor. Hence, four HATS risk groups could be defined: favorable (scores 0–1); intermediate (scores 2–3); poor (scores 4–5); and very poor (scores ≥ 6).

**Table 2:** Prognostic indicators for overall survival in the training cohort after allogeneic hematopoietic stem cell transplantation

	Univariate			Multivariate		
	HR	95% CI	*P*-value	HR	95% CI	*P*-value
Patient Factors						
Recipient ≥ 60 years	1.40	0.91, 2.17	0.127	—	—	—
Donor ≥ 35 years	0.73	0.54, 0.99	0.045	0.86	0.62, 1.19	0.365
Male recipient	1.50	1.10, 2.04	0.010	1.26	0.92, 1.72	0.150
Male donor	1.44	1.05, 1.97	0.025	1.31	0.95, 1.80	0.104
CMV positive recipient	0.94	0.67, 1.32	0.72	—	—	—
CMV positive donor	2.32	1.19, 4.55	0.014	2.02	1.02, 4.00	0.044
HCT-CI ≥ 2	2.44	1.53, 3.91	< 0.001	2.04	1.22, 3.40	0.006
Leukemia factors						
WCC ≥ 20,000 per microliter	1.06	0.78, 1.44	0.708	—	—	—
Secondary AML	2.33	1.52, 3.58	< 0.001	1.65	1.03, 2.65	0.036
Disease status at transplantatio			0.011			0.006
CR1	Ref	Ref	—	Ref	Ref	—
≥CR2	1.56	1.14, 2.13	0.005	1.71	1.22, 2.38	0.002
NR	2.36	0.75, 7.47	0.140	1.59	0.49, 5.14	0.441
Genetic risk group			< 0.001			< 0.001
CEBPA bZIP or CBF AML	Ref	Ref	—	Ref	Ref	—
Others	2.18	0.96, 4.95	0.063	2.93	1.19, 7.26	0.020
inv(3)/t(3;3) or KMT2A-r	4.84	1.96, 12.0	< 0.001	7.23	2.53, 20.7	< 0.001
-5/del(5q) or TP53mut	11.50	4.60, 28.9	< 0.001	11.70	3.96, 34.8	< 0.001
Low-intensity remission induction	2.44	1.68, 3.53	< 0.001	2.10	1.37, 3.21	< 0.001
HSCT factors						
Prior allogeneic HSCT	1.61	0.87, 2.96	0.129	—	—	—
Donor type			0.304			
Matched sibling	Ref	Ref	—			
Matched unrelated	0.98	0.65, 1.47	0.916	—	—	—
Mismatched unrelated	1.45	0.94, 2.26	0.094			
Haploidentical	1.20	0.79, 1.82	0.403			
Mismatched HSCT	1.30	0.95, 1.78	0.103	—	—	—
PBSC as graft source	1.08	0.76, 1.52	0.679	—	—	—
Reduced intensity conditioning	1.25	0.91, 1.70	0.164	—	—	—
PTCy-based GVHD prophylaxis	1.24	0.87, 1.75	0.232	—	—	—

*HR* hazards ratio for death, *CI* confidence interval, *Ref* reference group, *CMV* cytomegalovirus, *HCT-CI* hematopoietic cell transplantation-specific comorbidity index, *AML* acute myeloid leukemia, *ELN* EuropeanLeukemiaNet, *CR1* first complete remission, *NR* non-remission, *CEBPA bZIP* bZIP in-frame mutations in CEBPA, *CBF* core-binding factor, *KMT2A-r* KMT2A-rearranged, mut mutated, *HSCT* hematopoietic stem cell transplantation, *PBSC* peripheral blood stem cells, *PTCy* post-transplantation cyclophosphamide, *GVHD* graft-versus-host disease

**Table 3:** Prognostic score assignment based on overall survival in the training cohort after allogeneic hematopoietic stem cell transplantation

Risk factors	β coefficient	Score points
Donor CMV seropositivity	0.707	1
HCT-CI ≥ 2	0.694	1
Secondary AML	0.491	1
Non-CR1 at HSCT	0.568	1
Genetic risk group		
CEBPA bZIP or CBF AML	—	0
Others	0.790	1
inv(3)/t(3;3) or KMT2A-r	1.643	3
-5/del(5q) or TP53^mut^	2.221	4
Low-intensity remission induction	0.750	1

*CMV* cytomegalovirus, *HCT-CI* hematopoietic cell transplantation-specific comorbidity index, *AML* acute myeloid leukemia, *ELN* European LeukemiaNet; non-CR1: second complete remission or beyond, and non-remission; CEBPA bZIP: bZIP in-frame mutations in CEBPA; *CBF* core-binding factor, *KMT2A-r* KMT2A-rearranged; *mut* mutated

### HATS in the training cohort

 For the training cohort, there was a well-demarcated stepwise decrease in survivals when patients were divided into favorable (*N* = 50); intermediate (*N* = 313); poor (*N* = 81) and very poor (*N* = 22) risk groups (Fig. 2B). Pairwise log-rank tests with adjusted P-values showed significant survival differences between each pair of risk groups (Fig. 2C). For favorable, intermediate, poor and very poor risk groups, the respective median OS were not reached, not reached, 23 months and 7.3 months, and the respective 2-year OS were 92%, 78%, 50%, and 7.4% (Table 4); the respective 100-day cumulative incidences of grade 3–4 acute GVHD were 4.0%, 11%, 11%, and 9.1% (*P* = 0.295), and the respective 2-year cumulative incidences of moderate-to-severe chronic GVHD were 25%, 35%, 22%, and 30% (*P* = 0.166); the respective 2-year relapse-free survival rates were 92%, 72%, 38%, and 5.3% (*P* < 0.001), and the respective 2-year GVHD-free, relapse-free survival rates were 65%, 37%, 25%, and 0% (*P* < 0.001).

**Table 4:** Overall survivals (OS) according to HATS Risk Group in the training cohort

HATS risk group		HR	95% CI	*P*-value*	Median OS	95% CI	24-month OS	*P*-value†
Favorable		Ref	Ref	Ref	NR	-, -	92%	Ref
Intermediate		3.23	1.31, 7.94	0.011	NR	96, -	78%	0.007
Poor		7.90	3.14, 19.9	< 0.001	23 months	14, 87	50%	< 0.001
Very Poor		26.3	9.75, 70.9	< 0.001	7.3 months	5.1, 15	7.4%	< 0.001

*Cox proportional hazards regression

†Log-rank test. Overall P-value < 0.001; *P* < 0.05 is indicative significant differences in survival compared with reference (Ref) group

*HATS* Hong Kong Allogeneic HSCT Risk Score, *HR* hazards ratio for death, *CI* confidence interval

### Comparison of HATS with other prognostication models

Amongst the six existing models, AML-HCT-CR had the highest C statistic value (Fig. 1B) and was therefore chosen as the comparator. Compared with AML-HCT-CR, HATS provided a significantly better prediction of the risk of death following allo-HSCT (adjusted C-statistic: 0.662 *versus* 0.599, *P* = 0.001; AUC for risk of death within 2 years: 0.696 *versus* 0.628, *P* = 0.008) (Fig. 2D and E). Risk group re-distribution conceivably contributed to the improvement in survival prediction, with 78% of AML-HCT-CR low-risk cases re-classified as HATS intermediate-risk cases, and 32% of AML-HCT-CR intermediate-risk cases re-classified as HATS poor- or very poor-risk cases (Fig. 2F). Because HATS assigns a high score to genetic/cytogenetic changes that are largely based on ELN 2022, risk group re-distribution between HATS and ELN 2022 was also examined. The results showed that 71% of ELN 2022 favorable-risk cases were re-classified as HATS intermediate-risk cases; and 21% of ELN 2022 intermediate-risk cases were re-classified as HATS favorable-risk (6%), poor-risk (14%), or very poor-risk (1.4%) cases. Notably, 55% of ELN adverse-risk cases were re-classified as HATS favorable-risk (7%) and intermediate-risk (48%) cases; with only 46% of cases remaining as HATS poor- or very poor-risk cases (Fig. 2G). Therefore, the assessment of other relevant risk factors in HATS has significantly altered risk distribution based on genetics/cytogenetics as in ELN 2022, likely thereby improving the prognostic power.

### Validation cohort

**Fig. 3:**
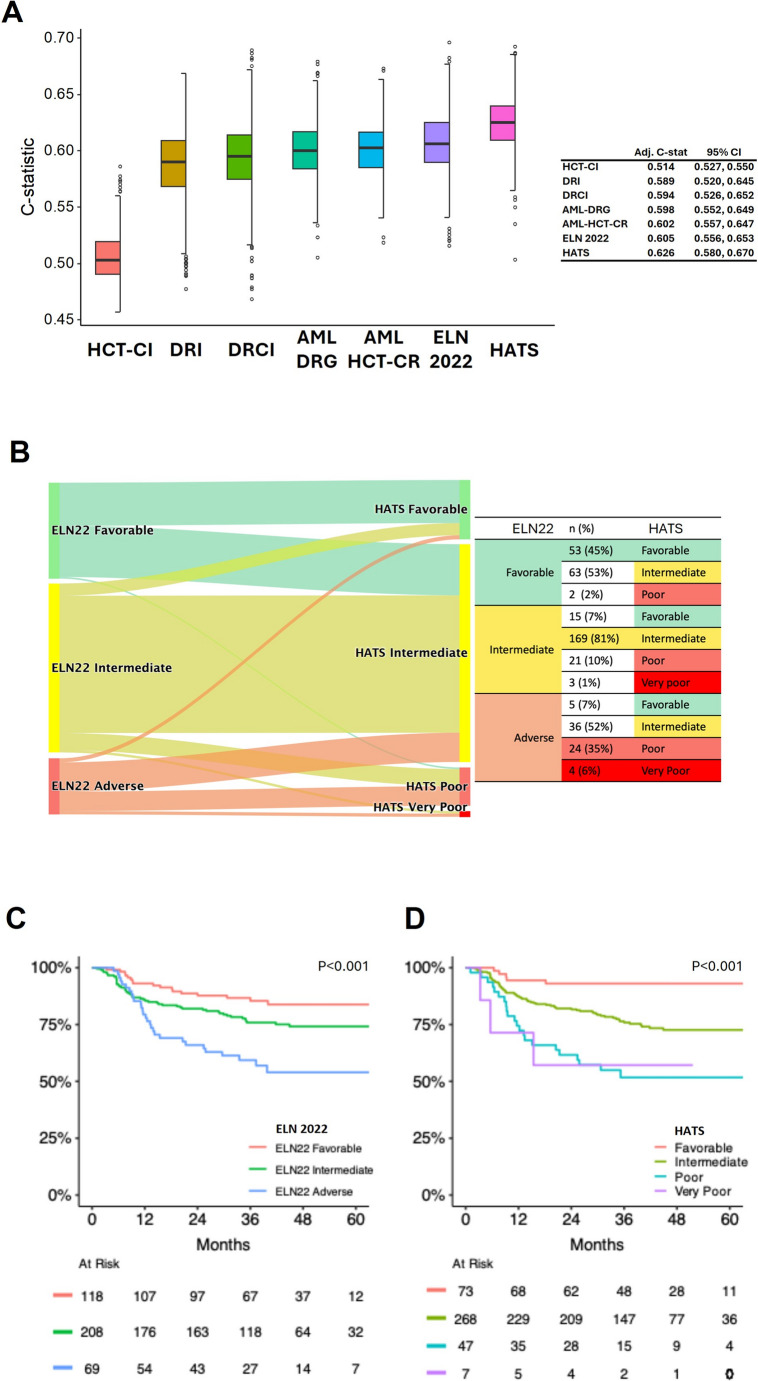
HATS in the validation cohort. **A**. Performance of different prognostication models by C-statistic in the external validation cohort. HATS achieved the highest C-statistic value. **B**. Re-stratification of risk groups from ELN 2022 to HATS in the validation cohort. **C**. Overall survival of the validation cohort by ELN 2022 classification. **D**. Overall survival of the validation cohort by HATS risk groups. ELN: European LeukemiaNet; HCT-CI: Hematopoietic Cell Transplantation-Specific Comorbidity Index; DRI: Disease Risk Index; DRCI: Disease Risk Comorbidity Index; AML-DRG: Acute Myeloid Leukemia-specific Disease Risk Group; AML-HCR-CR: AML Hematopoietic Cell Transplant-composite Risk; HATS: Hong Kong Allogeneic HSCT Risk Score; C: concordance; Adj. C-stat: adjusted C-statistic; CI: confidence interval.

Three hundred and ninety-five consecutive AML patients (men, *N* = 203; women, *N* = 192) with a median age of 41 (interquartile range, 31–52) years were analyzed (Supplementary Table 4). The validation cohort compared with the training cohort had significantly younger recipients (median age: 41 *versus* 51 years, *P* < 0.001) and donors (median age: 32 *versus* 38 years, *P* < 0.001); more male donors (66% *versus* 57%, *P* = 0.006); higher donor CMV seropositivity rate (86% *versus* 70%, *P* < 0.001); better ELN 2022 genetic profiles (favorable: 30% *versus* 21%, *P* < 0.001; adverse: 17% *versus* 32%, *P* < 0.001); more low-risk and fewer high-risk groups (*CEBPA* bZIP/CBF: 25% *versus* 13%, *P* < 0.001; -5/del(5q)/*TP53*^mut^: 3% *versus* 6%; *P* < 0.001); more haploidentical donors (77% *versus* 24%, *P* < 0.001); more peripheral blood HSCT (100% *versus* 75%, *P* < 0.001); fewer reduced-intensity conditioning regimens (14% *versus* 41%, *P* < 0.001); and no PTCy-based GVHD prophylaxis (0% *versus* 30%, *P* < 0.001) (Supplementary Table 5). Accordingly, the validation cohort mainly comprised low/intermediate-risk patients as assessed by existing prognostication models (Supplementary Table 6). HATS score ranged from 0 to 6, with 2 being the most common score (*N* = 167, 42%) followed by 3 (*N* = 101, 26%) and 1 (*N* = 67, 17%) (Supplementary Fig. 3). Increasing HATS scores were associated with progressively inferior OS by Kaplan-Meier analysis (Supplementary Fig. 3). Correspondingly, the validation cohort was stratified into HATS favorable: 18% (*N* = 73), intermediate: 68% (*N* = 268), poor: 12% (*N* = 47), and very poor: 2% (*N* = 7) groups (Supplementary Table 6). Similar to the training cohort although differing from it in many baseline characteristics, the validation cohort was best prognosticated by HATS, which achieved the highest C-statistic of 0.626 in comparison with the other six existing prognostic models, with ELN 2022 achieving the nearest at 0.605 (Fig. 3A). Risk group re-distribution was also evident, with 53% of ELN 2022 favorable-risk cases re-classified as HATS intermediate-risk cases, and 59% of ELN 2022 adverse-risk cases re-classified as HATS favorable-risk (7%) or intermediate-risk (52%) cases (Fig. 3B). Compared with AML-HCT-CR, HATS provided a significantly better prediction of the risk of death following allo-HSCT: the AUC for risk of death within 2 years was 0.619 versus 0.541 (*P* = 0.034) (Supplementary Fig. 4). Overall prediction error, measured by the IBS, was lower for HATS (0.150) compared with AML-HCT-CR (0.167). Over 0–24 months, the IBS was 0.092 for HATS and 0.110 for AML-HCT-CR, indicating better 2-year prediction accuracy for HATS, in addition to its superior discrimination. Furthermore, HATS outperformed ELN 2022 with superior discrimination of the favorable and intermediate groups. The 5-year OS of ELN 2022 favorable- and intermediate-risk groups were close at 84% (95% CI: 77–91%) and 74% (95% CI: 68–81%) (Fig. 3C); whereas the 5-year OS of the HATS favorable-risk and intermediate-risk groups were better separated at 93% (95% CI: 87–99%) and 73% (95% CI: 67–79%) (Fig. 3D). The 5-year OS of ELN 2022 adverse-risk group at 54% (95% CI: 42–69%) was comparable to those of HATS poor-risk and very poor-risk groups at 52% (95% CI: 39–69%) and 57% (95% CI: 30–100%) respectively (Fig. 3C and D). Notably, the poor-risk and very poor-risk groups in the validation cohort, as compared with the training cohort, were not as well separated, probably because of fewer number of patients in these risk groups.

## Discussion

In this study, we evaluated six existing prognostic models in a series of AML patients undergoing allo-HSCT. These models were selected because they are widely used, have been externally validated in allo‑HSCT, and could be accurately reconstructed from our available variables. With the same series as a training cohort, we performed multivariate analysis to define a more comprehensive panel of prognostic markers covering recipient, leukemia and donor related factors, thereby building a novel model HATS. The performance of HATS was compared with these models and shown to be superior to all six of them.

HATS assigns scores to six different clinicopathologic parameters. The highest scores are assigned to genetic/cytogenetic changes. Of the aberrations examined, inv(3)(q21;q26)/t(3;3)(q21;q26), *KMT2A* rearrangements, -5/del(5q) and *TP53*^mut^ were associated with the worst survivals and hence given the highest scores. The dismal outcome of AML with *TP53*^mut^ even with allo-HSCT had previously been reported [28, 29]. In fact, the negative prognostic impact of high-risk aberrations including − 5/del(5q), -7, -17/del(17p), and complex/monosomy karyotypes (CK/MK) could partly be mitigated by the absence of *TP53* mutations [30]. Similarly, we also found that patients with − 7, -17/del(17p), or CK/MK had intermediate risks in the absence of *TP53* mutations. However, -5/del(5q) in our cohort had outcome as poor as *TP53*^mut^. Secondly, donor CMV seropositivity was identified as an independent risk factor. This aligned with recent studies demonstrating that CMV-seropositive donors conferred higher mortality risk, primarily through increased non-relapse mortality (NRM) and relapse risk [31, 32]. However, donor CMV seropositivity should only be considered one independent adverse factor within HATS, whose impact needs to be balanced against other clinical and logistical considerations in donor-selection. The third risk factor is HCT-CI ≥ 2. Although HCT-CI has been extensively validated [5], its grouping of scores 1 and 2 together limits the discrimination in lower-risk patients. In fact, there was a substantial difference in NRM between patients with HCT-CI score of 0 and those with scores of 1 and 2 [5]. Hence, the lower threshold of ≥ 2 used in our study better discriminated patients with fewer comorbidities. The fourth risk factor is secondary AML, which has consistently been shown to have inferior post-HSCT survivals compared with *de novo* AML; [33, 34] with myeloablative conditioning reported to partially mitigate the difference [34]. In our study, secondary AML remained a significant risk independent of other factors including conditioning regimens in multivariate analysis. The fifth risk factor is non-CR1 at the time of allo-HSCT. A recent matched-pair analysis of allo-HSCT for AML showed that 5-year OS was significantly higher in CR1 than in CR2 (76.7% *versus* 59.3%, *P* = 0.026) [35]. Furthermore, MRD-negative patients in CR2 had outcomes similar to MRD-positive patients in CR1 after allo-HSCT for AML, underscoring the adverse impact of later remission status [36]. The sixth risk factor is low-intensity induction therapy. In adult AML patients unfit for intensive chemotherapy, venetoclax in combination with hypomethylating agents or low-dose cytarabine had acceptable outcomes [37, 38]. Although recent reports also showed similar survivals after allo-HCT for AML patients receiving low-intensity or intensive chemotherapy induction, these studies were limited by their retrospective nature and small study population [39–41]. Our finding that low-intensity induction was an independent risk requires further validation, especially in conjunction with MRD assessment to determine if suboptimal leukemia clearance by less intensive treatment might negatively impact on outcome after allo-HSCT.

The validation cohort in this study differed significantly from the training cohort in multiple clinicopathologic features, including a younger median age for donors and recipients, fewer ELN 2022 adverse-risk diseases, more favorable genetic/cytogenetic profiles, and more haploidentical HSCT. Despite these differences, HATS performed best in prognostication as compared with existing models, demonstrating its robustness and applicability in patients with different clinicopathologic and genetic landscapes.

With both the training and validation cohorts, a notable feature of HATS is its ability to identify a favorable group with excellent 2-year OS (training cohort: 92%; validation cohort:93%). It also identified a very high-risk group with a dismal 2-year OS of 7.4% in the training cohort. In the validation cohort, interpretation of a 2-year OS of 57% in the very high-risk group is limited by the small number of patients (*N* = 7) in this category. Combining the “poor” and “very poor” risk groups may therefore be appropriate in future external cohorts that include only a small number of patients with high‑risk features. Another limitation is that pre‑transplant MRD was available in only a small subset of patients and was assessed using heterogeneous methods, precluding robust statistical analysis and inclusion in the final model. We acknowledge this and consider systematic, standardized MRD collection a key priority for future refinements and external validations of HATS. Nonetheless, adoption of the HATS helps to better inform allo-HSCT outcome based on patient, and donor, disease, and treatment factors. Furthermore, the very poor outcome in the high-risk group suggests that therapeutic strategies other than allo-HSCT should be actively pursued in these patients.

Whether the HATS model is generalizable requires validation in more patient cohorts, particularly for factors not fully addressed in this study owing to sample limitations, including the independent negative impact of -5/del(5q) and low-intensity induction treatment on survival, the impact of active disease and high HCT-CI, and the significance of pre-HSCT MRD [42, 36, 17, 18]. 

We conclude that HATS represents a novel prognostication system that has outperformed existing models. Its specific advantages, including discrete discrimination between favorable- and intermediate-risk groups, and the identification of a very-high risk group, are highly relevant in management decisions and patient counselling.

## Supplementary Information

Below is the link to the electronic supplementary material.

Supplementary Material 1

## Data Availability

The datasets generated during and/or analysed during the current study are available from the corresponding author on reasonable request.
