# Supplementary material for: A novel prognostic scoring system HATS for acute myeloid leukemia patients undergoing allogeneic hematopoietic stem cell transplantation
Source: Ann Hematol. 2026 Apr 9;105(5):228. doi: 10.1007/s00277-026-06989-z (PMC13065535; doi:10.1007/s00277-026-06989-z)

# **Table of Contents**

[**Table of Contents** 1](#_Toc225100539)

[**Supplementary Table 1. Selection criteria for allogeneic hematopoietic stem cell transplantation, including definition of suboptimal measurable residual disease (MRD) response** 2](#_Toc225100540)

[**Supplementary Table 2. Prior induction regimens in the training cohort.** 3](#_Toc225100541)

[**Supplementary Table 3. Risk categorization by different prognostication models in the training cohort.** 4](#_Toc225100542)

[**Supplementary Table 4. Clinicopathologic features of the validation cohort of 395 patients with acute myeloid leukemia (AML) undergoing allogeneic hematopoietic stem cell transplantation (HSCT)** 5](#_Toc225100543)

[**Supplementary Table 5. Comparison of clinicopathologic features between the training and external validation cohorts.** 6](#_Toc225100544)

[**Supplementary Table 6. Risk categorization by different prognostication models in the validation cohort.** 7](#_Toc225100545)

[**Supplementary Figure 1. Overall survival by genetic subgroups in the training cohort.** 8](#_Toc225100546)

[**Supplementary Figure 2. Overall survival in the training cohort by HATS scores.** 9](#_Toc225100547)

[**Supplementary Figure 3. Overall survival in the validation cohort by HATS scores.** 10](#_Toc225100548)

[**Supplementary Figure 4. Time-dependent ROC (left) and AUC (right) of AML-HCT-CR and HATS in the validation cohort.** 11](#_Toc225100549)

# **Supplementary Table 1. Selection criteria for allogeneic hematopoietic stem cell transplantation, including definition of suboptimal measurable residual disease (MRD) response**

| 1. Intermediate or adverse risk by the latest ELN criteria in CR1 2. Non-remission to first induction 3. Relapsed AML achieving CR2 4. In patients with CBF AML and *NPM1*-mutated AML (without *FLT3*-ITD) in CR1 having a suboptimal MRD response  - Detectable MRD on peripheral blood after 2 cycles of chemotherapy. - <3-4 log reduction of MRD on bone marrow at the end of treatment. - MRD detected at ≥2% on bone marrow at the end of treatment. - >1 log rise in MRD during follow up. - Conversion of MRD from negative to positive during follow up. |
| --- |

ELN: European LeukemiaNet; CR1: first complete remission; CR2: second complete remission; CBF: core-binding factor; MRD: measurable residual disease

# **Supplementary Table 2. Prior induction regimens in the training cohort.**

| **Characteristic** | **Overall** N = 466 | **Intensive**  N = 405 | **Low-intensity^*^** N = 61 | **P-value** |
| --- | --- | --- | --- | --- |
| Age  Mean – years (SD)  Median – years (IQR) | 48.7 (11.4)  51 (16-68) | 48.4 (11.4)  50 (41-58) | 51.1 (11.2)  55 (47-59) | 0.046 |
| ELN 2022 risk      Favorable      Intermediate      Adverse | 97 (21%)  218 (47%)  151 (32%) | 97 (24%)  184 (45%)  124 (31%) | 0 (0%)  34 (56%)  27 (44%) | <0.001 |
| Relapsed disease | 156 (33%) | 127 (31%) | 29 (48%) | 0.012 |
| HCT-CI ≥2 | 34 (7.3%) | 31 (7.7%) | 3 (4.9%) | 0.60 |

SD: standard deviation; IQR: interquartile range; HCT-CI: Hematopoietic Cell Transplantation Specific Comorbidity Index; no.: European LeukemiaNet; CR1: first complete remission; CR2+: second complete remission or beyond; NR: non-remission; *: 33 (54%) received hypomethylating agents ± venetoclax and 28 (46%) received homoharringtonine based treatment.

# **Supplementary Table 3. Risk categorization by different prognostication models in the training cohort.**

| **Characteristic** | **Number (%)** |
| --- | --- |
| ELN 2022      Favorable      Intermediate      Adverse | 97 (21%)  218 (47%)  151 (32%) |
| HCT-CI  0  1-2  ≥3 | 368 (79%)  79 (17%)  19 (4%) |
| DRI  Low  Intermediate  High  Very high | 35 (7.5%)  387 (83%)  43 (9.2%)  1 (0.2%) |
| DRCI  Very low  Low  Intermediate-1  Intermediate-2  High  Very high | 34 (7.3%)  1 (0.2%)  374 (80%)  13 (2.8%)  39 (8.4%)  5 (1.1) |
| AML-DRG  Low  Intermediate  High | 290 (62%)  174 (37%)  2 (0.4%) |
| AML-HCT-CR  Low  Intermediate  High  Very high | 246 (53%)  162 (35%)  48 (10%)  10 (2.1%) |

ELN: European LeukemiaNet; HCT-CI: Hematopoietic Cell Transplantation-Specific Comorbidity Index; DRI: Disease Risk Index; DRCI: Disease Risk Comorbidity Index; AML-DRG: Acute myeloid leukemia-specific Disease Risk Group; AML-HCR-CR: AML Hematopoietic Cell Transplant-composite Risk.

# **Supplementary Table 4.** **Clinicopathologic features of the validation cohort of 395 patients with acute myeloid leukemia (AML) undergoing allogeneic hematopoietic stem cell transplantation (HSCT)**

| **Clinicopathologic parameters** | **Numbers** |
| --- | --- |
| Age  Recipient  Median – years (IQR)  ≥60 years – number (%)  Donor  Median – years (IQR)  ≥35 years – number (%) | 41 (31-52)  21 (5%)  32 (25-43)  175 (44%) |
| Male sex – number (%)  Recipient  Donor | 203 (51%)  261 (66%) |
| Donor CMV seropositivity – number (%) | 338 (86%) |
| HCT-CI – number (%)  0  1  ≥2 | 306 (77%)  55 (14%)  34 (9%) |
| White cell count at diagnosis  Median – per microliter (range)  ≥20,000 per microliter – number (%) | 15,750 (300-583,100)  164 (42%) |
| Secondary AML – number (%) | 24 (6%) |
| Disease status at transplantation – number (%)  CR/CRi  CR1  CR2  CR3+  NR | 345 (87%)  269 (68%)  69 (17%)  7 (2%)  50 (13%) |
| ELN 2022 risk classification – number (%)  Favorable  Intermediate  Adverse | 118 (30%)  208 (53%)  69 (17%) |
| Remission induction intensity – number (%)  Intensive  Low-intensity | 343 (87%)  52 (13%) |
| Prior allogeneic HSCT – number (%) | 0 (0%) |
| Donor type – number (%)  Matched sibling  Matched unrelated  Mismatched unrelated  Haploidentical | 57 (14%)  23 (6%)  11 (3%)  304 (77%) |
| HLA matching – number (%)  Matched  ≥1 HLA mismatched | 80 (20%)  315 (80%) |
| Hematopoietic stem cell source – number (%)  Peripheral blood  Bone marrow | 395 (100%)  0 (0%) |
| Conditioning intensity – number (%)  Myeloablative  Reduced intensity | 341 (86%)  54 (14%) |
| GVHD prophylaxis – number (%)  Conventional  PTCy-based | 395 (100%)  0 (0%) |

IQR: interquartile range; CMV: cytomegalovirus; HCT-CI: hematopoietic cell transplantation comorbidity index; CR: complete remission; CRi, complete remission with incomplete count recovery; NR, non-remission; HLA, human leukocyte antigen; MRD: measurable residual disease; GVHD, graft-versus-host disease; PTCy: posttransplantation cyclophosphamide

# **Supplementary Table 5. Comparison of clinicopathologic features between the training and external validation cohorts.**

|  | Training Cohort  (N=466) | Validation Cohort  (N=395) | P-value |
| --- | --- | --- | --- |
| Age – years, median (IQR)  Recipient  Donor | 51 (42-58)  38 (29-48) | 41 (31-52)  32 (25-43) | **<0.001**  **<0.001** |
| Sex – no. (%)  Male recipient  Male donor | 211 (45%)  265 (57%) | 203 (51%)  261 (66%) | 0.074  **0.006** |
| Donor CMV seropositivity | 328 (70%) | 338 (86%) | **<0.001** |
| HCT-CT ≥2 | 34 (7%) | 34 (9%) | 0.477 |
| Secondary AML | 43 (9%) | 24 (6%) | 0.085 |
| ELN 2022 – no. (%)  Favorable  Intermediate  Adverse | 97 (21%)  218 (47%)  151 (32%) | 118 (30%)  208 (53%)  69 (17%) | **<0.001** |
| Disease status at HSCT – no. (%)  CR1  Non-CR1 (≥CR2 and NR) | 307 (66%)  159 (34%) | 269 (68%)  126 (32%) | 0.490 |
| Genetic risk group – no. (%)  *CEBPA* bZIP or CBF AML  Others  Inv(3)/t(3;3) or *KMT2A*-r  -5/del(5q) or *TP53*^mut^ | 62 (13%)  342 (73%)  35 (8%)  27 (6%) | 98 (25%)  263 (67%)  22 (6%)  12 (3%) | **<0.001** |
| Low-intensity induction | 61 (13%) | 52 (13%) | 0.974 |
| Donor type – no. (%)  Matched sibling  Matched unrelated  Mismatched unrelated  Haploidentical | 177 (38%)  108 (23%)  67 (14%)  114 (24%) | 57 (14%)  23 (6%)  11 (3%)  304 (77%) | **<0.001** |
| PBSC as graft source | 349 (75%) | 395 (100%) | **<0.001** |
| Reduced intensity conditioning | 193 (41%) | 54 (14%) | **<0.001** |
| PTCy-based GVHD prophylaxis | 139 (30%) | 0 (0%) | **<0.001** |

IQR: interquartile range; no.: number of patients. ; CMV: cytomegalovirus; HCT-CI: hematopoietic cell transplantation-specific comorbidity index; AML: acute myeloid leukemia; ELN: European LeukemiaNet; CR1: first complete remission; CR2+: second complete remission or beyond; NR: non-remission; CEBPA bZIP: bZIP in-frame mutations in *CEBPA*; CBF: core-binding factor; *KMT2A*-r: KMT2A-rearranged; mut: mutated; HSCT: hematopoietic stem cell transplantation; PBSC: peripheral blood stem cells; PTCy: post-transplantation cyclophosphamide; GVHD: graft-versus-host disease

# **Supplementary Table 6. Risk categorization by different prognostication models in the validation cohort.**

| **Characteristic** | **Number (%)** |
| --- | --- |
| ELN 2022      Favorable      Intermediate      Adverse | 118 (30%)  208 (53%)  69 (17%) |
| HCT-CI  0  1-2  ≥3 | 306 (77%)  67 (17%)  22 (5.6%) |
| DRI  Low  Intermediate  High  Very high | 188 (48%)  150 (38%)  54 (14%)  3 (0.8%) |
| DRCI  Very low  Low  Intermediate-1  Intermediate-2  High  Very high | 180 (46%)  8 (2.0%)  140 (35%)  10 (2.5%)  53 (13%)  4 (1.0%) |
| AML-DRG  Low  Intermediate  High | 327 (83%)  24 (6.1%)  44 (11%) |
| AML-HCT-CR  Low  Intermediate  High  Very high | 324 (82%)  20 (5.1%)  7 (1.8%)  44 (11%) |
| HATS  Favorable  Intermediate  Poor  Very poor | 73 (18%)  268 (68%)  47 (12%)  7 (2%) |

ELN: European LeukemiaNet; HCT-CI: Hematopoietic Cell Transplantation-Specific Comorbidity Index; DRI: Disease Risk Index; DRCI: Disease Risk Comorbidity Index; AML-DRG: Acute myeloid leukemia-specific Disease Risk Group; AML-HCR-CR: AML Hematopoietic Cell Transplant-composite Risk: HATS: Hong Kong Allogeneic HSCT Risk Score

# **Supplementary Figure 1. Overall survival by genetic subgroups in the training cohort.**

|  |
| --- |

MDS: myelodysplasia; CK: complex karyotype; MK: monosomal karyotype

# **Supplementary Figure 2. Overall survival in the training cohort by HATS scores.**

|   Score 8  Score 7  Score 6  Score 5  Score 4  Score 3  Score 2  Score 1 |  |
| --- | --- |

# **Supplementary Figure 3.** **Overall survival in the validation cohort by HATS scores.**


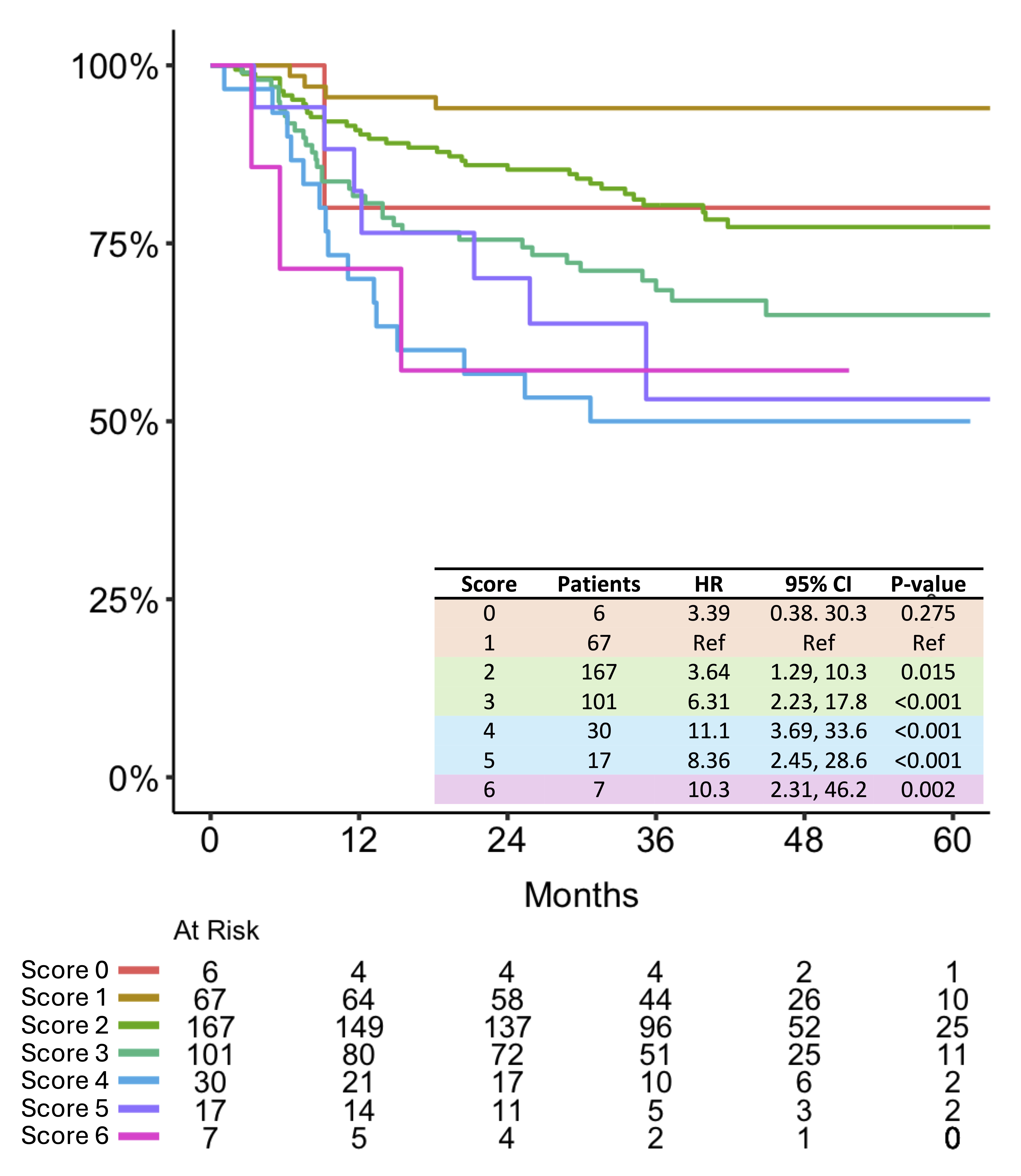


# **Supplementary Figure 4. Time-dependent ROC (left) and AUC (right) of AML-HCT-CR and HATS in the validation cohort.**


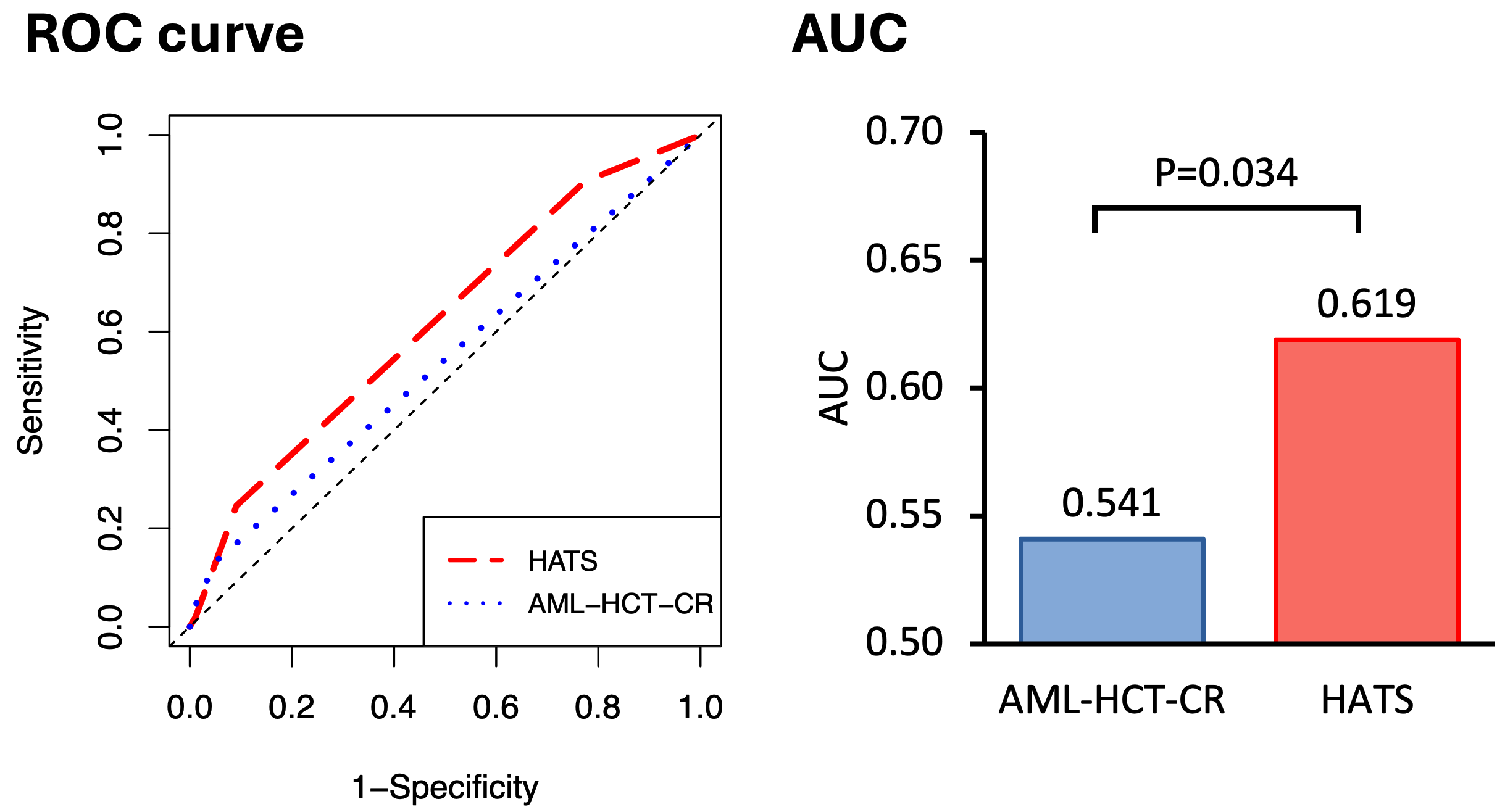

Supplement: Supplementary file 1 — Supplementary Material 1 [file 277_2026_6989_MOESM1_ESM.docx]
